# Reactive Oxygen Species (ROS) Regulates Different Types of Cell Death by Acting as a Rheostat

**DOI:** 10.1155/2021/9912436

**Published:** 2021-08-14

**Authors:** Gloria E. Villalpando-Rodriguez, Spencer B. Gibson

**Affiliations:** ^1^Research Institute in Oncology and Hematology, CancerCare Manitoba, University of Manitoba, Winnipeg, Manitoba, Canada; ^2^Department of Biochemistry and Medical Genetics, University of Manitoba, Winnipeg, Manitoba, Canada

## Abstract

Reactive oxygen species (ROS) are essential for cellular signaling and response to stress. The level of ROS and the type of ROS determine the ability of cells to undergo cell death. Furthermore, dysregulation of the antioxidant pathways is associated with many diseases. It has become apparent that cell death can occur through different mechanisms leading to the classifications of different types of cell death such as apoptosis, ferroptosis, and necroptosis. ROS play essential roles in all forms of cell death, but it is only now coming into focus that ROS control and determine the type of cell death that occurs in any given cell. Indeed, ROS may act as a rheostat allowing different cell death mechanisms to be engaged and crosstalk with different cell death types. In this review, we will describe the ROS regulatory pathways and how they control different types of cell death under normal and disease states. We will also propose how ROS could provide a mechanism of crosstalk between cell death mechanisms and act as a rheostat determining the type of cell death.

## 1. Introduction

Cellular reactive oxygen species (ROS) are tightly controlled to dictate different cell fates, such as differentiation or cell survival. When ROS are produced in excess, such as in cells under metabolic stress, this leads to cell death. This suggests that a “ROS rheostat” exists in cells controlling cellular survival. This rheostat controls ROS levels in the context of cellular microenvironmental signals ensuring that appropriate cellular functions are conducted. This is accomplished by ROS participating in cell signaling pathways, for example, during cell adhesion, host defense, or gene expression. When in excess, ROS may have deleterious effects on signaling and cellular damage leading to cell death.

Cell death was considered a passive event when cells become damaged or injured to a point that they disintegrate into cellular debris often termed necrotic cell death. It was not until genetic studies on *C. elegans* showed that many genes could control the amount of cell death, and physiological changes in cells undergoing cell death were reproducible and distinct [1, 2]. Apoptosis was the first cell death type to be described in this way, where cell membranes become blebbed and chromatin becomes condensed. This form of cell death was implicated in many physiological and pathological conditions such as immune system development and cellular homestasis [3, 4]. Over time, other forms of cell death were described under different physiological conditions such as iron-mediated cell death, ferroptosis, and autophagy- (self-eating-) induced cell death. Similar to apoptosis, they are regulated by distinct genes; for instance, some of the key genes involved in apoptosis regulation are caspases, TP53, FAS, BCL-2, and BAX. Genes that regulate ferroptosis include GPx4, Nrf2, LSH, TFR1, and SLC7A11. Necroptosis is regulated by LEF1, RIP1, and RIP3, and finally, autophagy regulations are regulated by genes ATG5, ATG7, DRAM3, and TFEB [5, 6].

The different types of cell death induce specific cell signaling pathways. Nevertheless, there are common features among these cell death pathways. One predominant feature is the reliance on ROS signaling and control. ROS produced by cells under stress, or cells with reduced antioxidant capacity, can determine whether the cell survives or dies, and the type of cell death mechanism engaged. This ability of ROS to act as a rheostat determining not only cell death but the type of cell death is only now coming into focus under pathological or physiological conditions. This review will explore the importance of ROS and its signalling on different types of cell death and how ROS acts a rheostat to determine different types of cell death.

## 2. Regulation of ROS Production and Antioxidants

Reactive oxygen species (ROS) is a type of unstable molecule that contains oxygen and easily reacts with other molecules in a cell [7]. ROS include reactive molecule derivatives of oxygen (nonradicals) and also oxygen-centered radicals (free radicals). Free radicals and nonradicals can react with each other to produce more free radicals and nonradicals; for instance, two superoxide anions (O_2_^•−^) can react to form hydrogen peroxide (H_2_O_2_), a nonradical (2O_2_^•−^ + 2H_2_⟶H_2_O_2_ + O_2_). In turn, hydrogen peroxide can break down in the presence of transition metal ions to produce hydroxyl radical HO^−^, the most reactive and damaging of all oxygen free radicals [4, 8, 9]. Other oxygen-derived free radicals are peroxide ion (O_2_^•2−^), perhydroxyl radical (HO_2_^•^), alkoxyl radical (RO^•^), and peroxyl radical (ROO^•^). Singlet oxygen (^1^O_2_) and hypochlorous acid (HOCL) are other nonradical derivatives of oxygen [10, 11].

**Figure 1:**
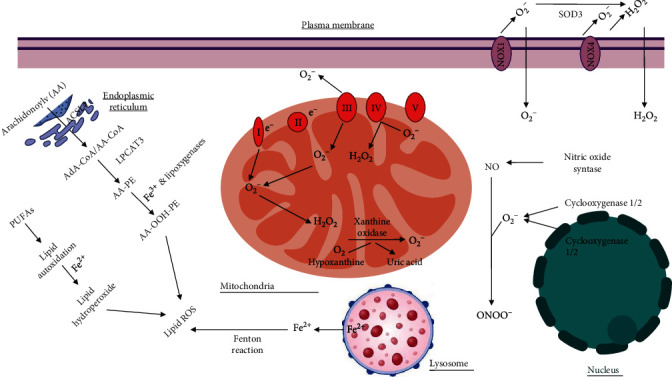
Subcellular localization of reactive oxygen species (ROS). The plasma membrane contains NOX enzymes that generate superoxide and hydrogen peroxide. The mitochondrial electron transport chain generates superoxide and hydrogen peroxide at several locations within the mitochondria. Lysosome releases reactive iron that generates lipid ROS. The nucleus generates superoxide through cyclooxygenases. Finally, the endoplasmic reticulum generates lipid ROS.

Under physiological conditions, ROS are generated by numerous sources including mitochondria respiratory chain (the major source), NADPH oxidases, xanthine oxidases, lipoxygenases, nitric oxide synthases, and cyclooxygenases (Figure 1). Ninety percent of ROS are generated when electrons escape from the mitochondrial electron transport chain (ETC). The ETC is composed of transmembrane protein complexes (I-IV) and ubiquinone and cytochrome c (electron transfer carriers); when these complexes are assembled, together with complex V (F_1_F_0_ATP synthase), the oxidative phosphorylation occurs resulting in ATP production. There are two electron transport pathways in the ETC: complex I/III/IV, with NADH as substrate and complex II/III/IV, with succinic acid as substrate. The electrons in the ETC leak out and interact with oxygen to produce superoxide or hydrogen peroxide. CI and CIII, especially CI, are considered to be the main sites of ROS production in mitochondria. There are 11 sites within the ETC where superoxide or hydrogen peroxide are produced. In the matrix, at sites I_F_ (FMN site) and I_Q_ (CoQ binding site), ROS are produced during the transfer of electrons from NADH to CoQ in CI. CII produces ROS at site II_F_ associated with succinate dehydrogenase. CIII transfers electrons through the Q-cycle; in this process, ubisemiquinone (QH^−^) of the Q_o_ site carrying a single electron can move freely in CIII, directly leaking the single electron to O_2_, forming ROS through a nonenzymatic reaction. Then, the formed ROS can be released into both the matrix and the intermembrane space; here, superoxide dismutase converts superoxide into hydrogen peroxide, which freely disperses through the outer membrane of mitochondria. It has been found that superoxide can also translocate to the cytosol through anion channels [8, 9, 12, 13]. Interestingly, ROS production by the mitochondria ETC causes oxidation of cysteine residues in mitochondrial proteins, modifying their function. For instance, cysteine residues in the 51 and 75 kDa subunits of the hydrophilic arm of complex I are sites of S-oxidation (oxidation on thioether moieties of cysteine to form sulfoxides), resulting in decreased complex I activity by limiting proton-motive force reduction and electron flow through the respiratory chain, which could become irreversible upon further oxidation [14]. The electron transport chain from other membranes in the cell (endoplasmic reticulum and plasmatic and nuclear membranes) also produce ROS but at a small scale compared to mitochondria [15–17]. ETC and proton-motive force regulates ROS accumulation, but how this controls the ROS rheostat remains unclear.

As mentioned before, several enzymes are responsible for ROS production. The most important, NADPH oxidase (NOX), catalyzes the process called respiratory burst. There are seven NOX family members: NOX 1-5 and dual oxidases 1 and 2 (DUOX 1 and 2); upon activation, they reduce dioxygen to superoxide anion using NADPH or NADH as an electron donor. Despite the fact that NOX members have a similar structure and enzymatic activity, they differ in their activation mechanism. For instance, NOX 1-4 require p22^phox^, NOX 1 and 3 need NOXO1, and the small GTPase Rac subunit is associated with NOX 1 and 2. In addition, NOX5 and DUOX 1-2 are activated by calcium and do not require any other subunit [18]. The DUOX enzymes have a double function; they are ROS-generating enzymes and use H_2_O_2_ to carry out oxidations of other substrates using their peroxidase domain [19]. In neutrophils, NOX activity results in the release of superoxide that plays a bactericidal role. In nonimmune cells, NOX activity plays a role in proliferation, migration, cell adhesion, and growth, and in the nucleus, it plays a role in gene expression [7, 20–24]. Here, GSH donates a hydrogen atom from water oxidizing GSSG. Another scavenger vitamin is ascorbic acid (vitamin C).

Antioxidants are responsible for ROS elimination or prevention of ROS formation to avoid damaging oxidative stress. The antioxidant systems can be divided as enzymatic and nonenzymatic (Table 1). The first one consists mainly in superoxide dismutases, catalase, glutathione peroxidases, peroxiredoxins, and thioredoxins. Nonenzymatic antioxidants are molecules that act by directly quenching free radicals or by radical scavenging; this include but are not limited to vitamins E, C, and A; glutathione; and uric acid [8, 10, 25].

### 2.1. Enzymatic Antioxidants

Superoxide dismutase (SOD) enzyme is the most important one that protects the cells against superoxide anion. According to cation type and cellular localization, there are three SODs: SOD1 or copper/zinc Cu/ZnSOD, localized mainly in the cytoplasm (but also found in mitochondria and nucleus); SOD2 or manganese MnSOD, localized in the mitochondrial matrix; and extracellular SOD3 or copper/zinc SOD. SODs catalyze superoxide dismutation into hydrogen peroxide using copper/zinc or manganese as cofactors that continuously shift between reduced and oxidized forms in the active site of the enzymes. SOD activity is important not only because it prevents the accumulation of superoxide anion but also because it prevents its reaction to nitric oxide (NO). When these two molecules react, there is not only the production of peroxynitrite, a strong oxidant, but also the inactivation of NO which has anti-inflammatory and anticoagulant properties. Finally, superoxide dismutation by SODs produces hydrogen peroxide which, as mentioned before, is an important signaling molecule [10, 26, 27]. SODs catalyze transformation of superoxide anion by dismutation into hydrogen peroxide. In turn, catalase decomposes hydrogen peroxide into water and oxygen. Catalase contains four identical subunits of 62 kDa, each subunit containing four distinct domains and one prosthetic heme group. One domain has a distal histidine essential for catalase reaction, the second domain has a hydrophobic core that confers its tridimensional structure, the third domain has a tyrosine residue for heme group binding, and the last domain is an *α*-helical domain for NADPH binding. Catalase has a nuclear origin and contains a peroxisome-targeting signal sequence KANL (lysine-alanine-asparagine-leucine) that imports catalase monomers to the perixosomes where tetramerization and heme addition occur. The hydrogen peroxide decomposition occurs in two reactions: in the first reaction, the catalase heme group is oxidized by one molecule of hydrogen peroxide into a hypervalent iron intermediate compound I (an oxoferryl porphyrin cation radical) with concomitant production of water, and in the second reaction, compound I is reduced by a second hydrogen peroxide molecule to its resting state generating two molecules of water and oxygen [28, 29].

Catalase is not the only antioxidant enzyme that decomposes hydrogen peroxide into water. Glutathione peroxidases (GPxs) belong to a family of enzymes that catalyze the reduction of hydrogen peroxide into water and organic hydroperoxides into alcohols using glutathione as reductant. There are eight human GPxs. GPx1-4 and GPx6 are selenoproteins, whereas GPx5, GPx7, and GPx8 have a cysteine in the catalytic site [30, 31]. GPx4 is widely expressed and differs in its structure and substrate specificity compared to the other family members. This monomer, can react not only with hydrogen peroxide but also with a wide range of lipid hydroperoxides, including those derived from cholesterol and cholesteryl esters using GSH as reducing substrate, though it can also use protein thiols as reductants [32].

Thioredoxins are enzymes that reduce oxidized proteins through the TXR active site that contains a specific and highly conserved motif with two residues of cysteine. There are two isoforms: TRX1, which is localized in the cytosol and nucleus, and TRX, which is found in mitochondria. Thioredoxin reductase enzymes continuously reconvert the oxidized TXR form into the reduced form. Peroxiredoxins reduce hydrogen peroxide by cycling between oxidation and reduction reactions thanks to their enzymatic active site constituted by cysteine amino acids that metabolize H_2_O_2_. During these redox reactions, the cysteine recycling is mediated by GSH, ascorbic acid, or sulfiredoxins [22].

### 2.2. Nonenzymatic Antioxidant

GSH is a nonenzymatic antioxidant, the most abundant nonprotein thiol GSH. Its reduced form is tripeptide g-glutamil-cysteinyl-glycine, and its second form is the disulfide-oxidized GSSG. It localizes in the cytoplasm, in the outer mitochondrial membrane, within the endoplasmic reticulum, and in bile and plasma. GSH is synthesized in the cytosol in two reactions. The first one is the rate-limiting reaction that generates *γ*-glutamylcysteine (*γ*-Glu-Cys) from cysteine and glutamate by the enzyme glutamate cysteine ligase. In the second reaction, glycine is added to the C-terminus of *γ*-Glu-Cys to generate reduced glutathione (GSH) by the enzyme glutathione synthetase. GSH has several functions, but when acting as an antioxidant, its function is accomplished by glutathione peroxidase (see above) catalyzed reactions. Here, GSH donates a hydrogen atom from water and the oxidized GSSG. GSSG in turn is reduced back to GSH by GSSG reductase at the expense of NADPH, forming a redox cycle. GSH can also react directly to oxygen radicals by a radical transfer process giving place to the GSH thiol radical and eventually to GSSG [33–35]. Other nonenzymatic antioxidants include vitamins. For instance, the scavenger *α*-tocopherol (vitamin E) is a lipophilic compound known as a “chain-breaking antioxidant” that protects membranes from oxidation by intercepting lipid peroxyl radicals, preventing the propagation step in the lipid peroxidation process. By doing so, *α*-tocopherol becomes a tocopheroxyl radical, a stable radical insufficiently reactive to participate in lipid peroxidation reactions [8, 36, 37]. Another scavenger vitamin include ascorbic acid (vitamin C). This hydrophilic molecule acts as an antioxidant by donating electrons, thus acting as a reducing agent that prevents the oxidation of other molecules. By donating electrons, ascorbic acid is oxidized, transforming itself into a free radical called ascorbyl radical; however, this radical is stable and almost unreactive. Also, ascorbic acid can regenerate a tocopheroxyl radical by reducing it back to *α*-tocopherol [9, 38].

The balance between prooxidant and antioxidant regulatory mechanisms within a cell determines whether it survive or dies. Under physiological conditions, the production of reactive oxygen species (ROS) is counterbalanced by their elimination and/or prevention of formation to maintain a steady-state (stationary) ROS level. This maintains cellular homeostasis, allowing ROS to act as signaling molecules to accomplish physiological functions. When this balance is lost, favouring enhanced ROS levels (through increased ROS production or decreased antioxidants), oxidative damage can occur on proteins, lipids, DNA, nucleic acids, and other macromolecules, leading to functional disturbances and eventually to cell death [11, 39, 38].

Oxidative stress can be classified depending on its intensity. Under physiological conditions, oxidative stress is called oxidative eustress, while the exposure to supraphysiological oxidative challenge is called oxidative distress [38]. Another classification of oxidative stress also based on its intensity has been proposed. In this classification, there are four zones: (1) basal oxidative stress (BOS), (2) low-intensity oxidative stress (LOS), (3) intermediate intensity oxidative stress (IOS), and (4) high-intensity oxidative stress (HOS) [9]. By setting a ROS rheostat level, cellular functions can adapt to microenvironmental signals in favour of cell survival or in favour of a specific cell death pathway as described in the following sections.

## 3. ROS Signaling

Beyond regulating the level of ROS within the cell using antioxidant or oxidative pathways, ROS induces a wide range of signals within the cell. One of these major signals is the ability of ROS to activate transcription factors. A study reported that production of hydrogen peroxide by xanthine oxidase regulates gene expression of c-jun and c-myc [7]. Hydrogen peroxide, acting as a signaling molecule, has been shown to induce gene expression, through activation of transcription factors c-jun, egr-1, and JE [40, 41]. ROS, in particular hydrogen peroxide, also activates transcription factor NF-*κ*B. Degradation of the NF-*κ*B inhibitory subunit (I*κ*B) is necessary for NF-*κ*B activation. IKK phosphorylates I*κ*B leading to its ubiquitination and degradation. It has been shown that IKK is S-glutathionylated by ROS inactivating its kinase activity [42, 43]. The kinase upstream of IKK, MEKK1, is also regulated by ROS. MEKK1 is a redox-sensitive kinase that can also be glutathionylated leading to its inactivation. Finally, ROS can block the ubiquitination and degradation of I*κ*B through inhibition of UBcl2 [44, 45]. This activation of NF-*κ*B leads to upregulation of genes associated with cell survival and activation of a negative feedback loop where antioxidant genes such as SOD family members are upregulated (for more detailed information on NF-*κ*B in oxidative stress, refer to Lingappan) [46].

Besides transcription factors, ROS can regulate several signaling pathways affecting many cellular functions and ultimately influencing cell survival or cell death. The MAPK signaling pathway can be activated by ROS. This pathway that includes MAPKs Erk, JNK, and p38 play important roles in cell growth, differentiation, development, cell cycle, and cell survival. The MAPK pathways are often activated by growth factor activation of receptor tyrosine kinases. This leads to the activation of small G proteins such as RAS that leads to MAPK pathway activation. ROS has been shown to activate receptor tyrosine kinases without binding of the ligand and inactivate dual-specificity phosphatase that negatively regulates MAPKs [47]. The JAK/STAT pathway can be activated by ROS oxidation of glutathione [48]. In addition, ROS can allow ASK1 oligomerization and autophosphorylation through oxidizing thioredoxin which inhibits the activation of ASK1. Similar to Erks, phosphatases may be inactivated by ROS leading to prolonged activation of JNK. MAPK p38 is also activated by ROS mediated by MAPK kinase kinase such as ASK1 or MEKK1-4 [49, 50]. The PI3K-Akt signaling pathway, which participates in functions such as protein synthesis, cell cycle progression, proliferation, and cell death, can also be regulated by ROS. On one hand, ROS directly activate PI3K, amplifying its downstream signaling. On the other hand, ROS can inactivate PTEN, which negatively regulates the synthesis of PIP3, inhibiting the activation of Akt, via oxidizing cysteine residues within the active center [51].

Finally, there are regulatory systems responsible for adaptation in response to oxidative stress. The nuclear factor erythroid 2-related factor 2 (Nrf2) pathway is one of them. The Nrf2 protein is ubiquitously expressed, and it is found in the cytosol interacting with its suppressor E3 ligase adapter Kelch-like ECH-associated protein 1 (KEAP1). This interaction happens by binding between the kelch domain of KEAP1 and the Nrf2 second domain (Neh2) (seven domains in total) at two amino acid sequences DLG and ETGE [52–54]. In normal conditions, KEAP1 presents NRF2 for ubiquitination by the E3 ligase complex formed by Cullin3 and RBX1 proteins (CUL3/RBX1), resulting in subsequent NRF2 proteasomal degradation. Additionally, Nrf2 can be degraded by ubiquitination, independent of Keap1, and by phosphorylation of the Nrf2 Neh6 domain [55, 56]. Under redox-challenging conditions, where high levels of ROS induce glutathionylation and alkylation of macromolecules, Nrf2-KEAP1 is dissociated and Nrf2 is translocated to the nucleus. Alteration in KEAP1 cysteine residues leads to the activation of Nrf2. The Nrf2 Neh5 domain has a redox-sensitive nuclear export signal, and once in the nucleus, Nrf2 functions as a transcription factor, and the Neh1 domain enables Nrf2 binding to the antioxidant region elements (AREs: enhancer sequences in the regulatory region of Nrf2 target genes) leading to the expression of antioxidant genes like glutathione peroxidases and stress response iron genes, like for instance, heme-oxygenase 1 [57–59].

Based upon the transcriptional signatures that ROS generates, this could lead to (1) execution of physiological functions such as cell adhesion, migration, and growth; (2) cell survival and proliferation; or (3) cell arrest and cell death. Many ROS-mediated signaling pathways activate cell survival mechanisms such as upregulation of anticell death BCL-2 family members (via NF-*κ*B) or increased expression of antioxidant enzymes that prevents buildup of ROS-generated cell damage, for instance, through the Nrf2 pathway. Conversely, ROS-mediated signaling could activate procell death pathways such a JNK signaling [13, 43, 53]. The balance between this competing ROS signaling pathways will set the rheostat for cellular homeostasis and will determine how a cell will survive or which type of cell death a cell undergoes.

## 4. ROS in Different Types of Cell Death

**Figure 2:**
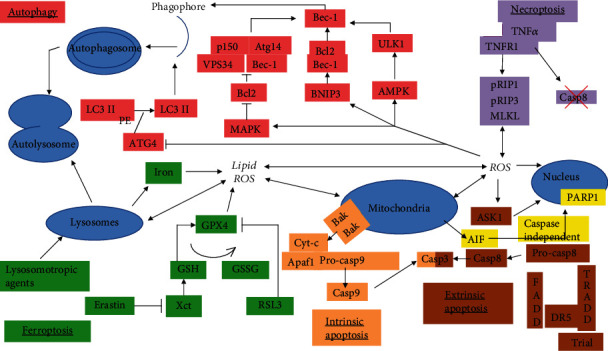
Role of reactive oxygen species (ROS) in different types of cell death signaling. ROS contributes to autophagy-induced cell death through activation of BNIP3, AMPK, and MAPK and inactivation of ATG4. ROS also induces apoptosis through mitochondrial damage, ASK1 activation, and PARP activation. Ferroptosis is regulated by lipid ROS leading to lysosome disruption, mitochondrial damage, and increased ROS. Finally, RPS contributes to necroptosis through activation of the MLK1 complex.

When cells die, there is a corresponding increase in ROS from different sources within a cell. During an increase in ROS, damage to cellular organelles such as the mitochondria occurs. Also, ROS produced by nitroxide synthase causes damage to the plasma membrane contributing to cell death. In addition, antioxidant pathways can also be inhibited leading to increased levels of ROS and cell death. At the same time, ROS triggers signaling pathways that contribute to cell survival such as transcription factor NF-*κ*B that increases the expression of antiapoptotic proteins. This balance will determine whether the cell dies or survives. However, how ROS regulate the different types of cell death is only recently coming into focus. For the purpose of this review, four types of cell death will be described (apoptosis, ferroptosis, autophagy, and necroptosis) and the role of ROS in these types of cell death (Figure 2) will be discussed:

### 4.1. Apoptosis

Apoptosis was the type of programmed cell death that was first described. During apoptosis, three major events take place: activation of protease, degradation of DNA, and phagocytization of apoptotic bodies by neighbour cells. Depending on the proteases activated, we can distinguish two different types of apoptosis: caspase dependent and caspase independent. Caspase-dependent apoptosis can be triggered by two pathways depending on the nature of the inducer agent, that is, by extracellular (extrinsic pathway) or intracellular (intrinsic pathway) perturbations.

The apoptotic extrinsic pathway is initiated by a death receptor and its ligand or by a dependence receptor and the drop on the levels of its ligand. Death receptors contain in their intracellular region a domain called death domain that allows the formation of the death-inducing signaling complex (DISC). The best studied death domains are Fas, TNFR1, and DR4-5, and the ligands involved in this pathway are Fas ligand, TNF-*α*, and TNF-related apoptosis-inducing ligand (TRAIL). DISC is composed of the death receptor, an adaptor protein like the Fas-associated death domain (FADD) or the TNF receptor 1-associated protein (TRADD), and procaspase 8. This last element suffers autoproteolitic cleavage that results in the activation of caspase 8 which in turn will activate executioner caspase 3 [60–63]. Even though intracellular ROS have been shown to activate intrinsic apoptosis (described below), there is evidence that ROS also participate in the execution of extrinsic apoptosis; even more, it has been found that ROS can induce both extrinsic and intrinsic pathways at the same time. In a study, HL-7702 cells treated with matrine showed an increase in ROS levels and lipid peroxidation as well as a decrease in SOD and GSH activity in a dose-dependent manner. Matrine also induced upregulation of Keap1 expression and downregulation of cytosolic and nuclear Nrf2, and it also inhibited the expression of Nrf2 downstream targets, heme-oxygenase and quinone reductase NQO1, and promoted the KEAP1/Nrf2 complex formation. Mitochondrial membrane potential was decreased, and cytochrome c was released from the mitochondria to the cytosol. Besides the intrinsic pathway, matrine also increased Fas death receptor expression and caspase 8 activation. In addition, antioxidant N-acetylcysteine (NAC) partially reduced matrine-induced apoptosis [64]. It was also shown that TNF-*α*-sensitive mesangial cells stimulated by the death receptor ligand TNF-*α* undergo apoptosis through increased superoxide anion, whereas its downstream compounds hydrogen peroxide and peroxynitrite remained unchanged. This was demonstrated by using the superoxide anion scavenger Tiron, the hydrogen peroxide scavengers GSH and catalase, and the peroxynitrite scavenger uric acid. Results showed no effect from pretreatment with GSH, catalase, or uric acid on cell death; only Tiron was able to reduce all apoptotic markers. This was further confirmed by transient transfection with SOD2 plasmid that significantly reduced the percentage of apoptotic cells, whereas catalase transfection had no effect on cell death. Pretreatment with both catalase and uric acid had no effect on TNF-*α* induced cell death [65]. Thus, ROS is involved in the extrinsic apoptotic pathway.

As mentioned before, the apoptotic intrinsic pathway is initiated by intracellular stress such as DNA damage, endoplasmic reticulum stress, and oxidative stress. These stress signals generally activate proapoptotic proteins BAK and BAX, belonging to the BCL-2 family of proteins. These two proteins mediate mitochondrial outer membrane permeabilization by forming pores across the outer mitochondrial membrane. Oxidative stress is a factor that enhances the opening of the mitochondrial permeability transition pore (mPTP). A transient opening may occur under physiological conditions; however, sustained opening of mPTP induces cell death by increasing oxidant stress, causing ATP depletion, and/or by triggering matrix swelling and subsequent rupture of the outer mitochondrial membrane. This permeabilization leads to the release of apoptotic molecules like apoptosis-inducing factor (AIF), SMAC/DIABLO, or cytochrome c. Cytosolic cytochrome c binds to the apoptotic protease-activating factor-1 (APAF-1) that undergoes oligomerization and recruits procaspase 9 forming the apoptosome. Procaspase 9 within the apoptosome is activated and liberated from the complex; caspase 9 then activates the executioner caspases 3 and/or caspase 7 [62, 66–68]. The intrinsic apoptotic pathway is the most studied form of cell death that requires increased mitochondrial ROS. This mitochondrial ROS is generated by dysfunctional oxidative phosphorylation mediated in part by NOX. It has been shown, in an *in vivo* model, that angiotensin II induced sustained activation of calcium/calmodulin- (Ca^2+^/CaM-) dependent protein kinase II due to NOX oxidation, resulting in myocardial apoptosis. Moreover, transgenic mice p47^−/−^ (unable to assemble the ROS-producing complex NADPH oxidase) did not show apoptotic markers after angiotensin II treatment; however, direct addition of hydrogen peroxide caused apoptosis, showing that the resistance to induce apoptosis is due to NOX inability to produce ROS [69]. In addition, inhibition of thioredoxin reductase leads to increased ROS and apoptosis [70]. Another source of ROS comes from the deregulation of the antioxidant pathways leading to apoptosis. In thymocytes, glucocorticoid methylprednisolone induced production of peroxides and depletion of GSH prior to an increase of intracellular calcium and later DNA fragmentation, as shown by the oligonucleosomal-length fragments [71]. Another study found that artesunate treatment of doxorubicin-resistant T leukemia cells resulted in an accumulation of hydrogen peroxide followed by caspase-dependent apoptosis via the intrinsic pathway as confirmed by the release of cytochrome c from mitochondria, the activation of caspase 9 and caspase 3, and DNA fragmentation. Moreover, pretreatment with NAC not only decreased hydrogen peroxide levels but also significantly blocked artesunate-induced apoptotic cell death [65]. Accumulation of superoxide anion in vascular endothelial ECV304, treated with *Vibrio vulnificus* cytolysin, induced release of cytochrome c, caspase 3 activation, and DNA fragmentation. In addition, the superoxide anion scavenger TEMPO successfully blocked its production, totally blocked cytochrome c release, abolished caspase 3 activation, and completely inhibited apoptosis [66]. Finally, lysosome-disrupting agents lead to lysosomal membrane permeabilization (LMP), causing increased ROS production followed by mitochondrial dysfunction and apoptosis [72]. This indicates a central role of ROS in the intrinsic apoptotic pathway.

Mitochondrial dysfunction due to ROS can induce the release of mitochondrial AIF resulting in its translocation to the nucleus where it causes DNA condensation and apoptosis through caspase-independent mechanisms [73]. The effect of oxidized alpha-linolenic acid-rich oils was analyzed in neuronal SH-SY5Y cells. After 3 hours of treatment with oxidized perilla and linseed oil (oxidized by heat), cells presented increased ROS levels followed by loss in mitochondrial membrane potential. Annexin V and PI staining showed that the oxidized oil treatment induced apoptosis, and pretreatment with antioxidant drug N-acetylcysteine (NAC) decreased apoptosis. Even more, oxidized oil treatment also induced release of mitochondrial AIF and an increase in nuclear truncated AIF (tAIF) expression, showing activation of caspase-independent apoptosis. Blocking ROS accumulations with NAC results in restoring mitochondrial AIF levels but had no effect on caspase-3-activated levels [74]. This suggest that ROS plays a role in both caspase-dependent and independent apoptosis.

### 4.2. Ferroptosis

The cell death mechanism called ferroptosis is a newly described regulated cell death characterized by the iron-dependent intracellular accumulation of ROS and lipid peroxidation products. From a biochemical, morphological, and genetic perspective, ferroptosis is different from other regulated cell death types. During ferroptosis, cells are rounded-up and detached and mitochondria size is smaller than the normal, with increased mitochondrial membrane density and reduction/vanishing of mitochondria crista and outer mitochondrial membrane rupture, similar to apoptosis. Unlike apoptosis, normal nuclear size and nonchromatin condensation is observed [75, 76].

Ferroptosis was first characterized by treatment of cells with two small molecules, Ras Selective Lethal (RSL) and erastin that were developed to be selectively lethal to cells expressing oncogenic mutant Ras. Erastin was shown to inhibit the mitochondrial voltage-dependent anion channel 2 and 3 (VDAC2 and VDAC3) and cysteine/glutamate antiporter or system xCT. The xCT system is an antiporter composed of the transmembrane transporter protein SLC7A11 and the single-pass transmembrane regulatory protein SLC3A2, both linked by a disulfide bridge. It imports extracellular cysteine and exports intracellular glutamate. Cysteine import is necessary for GSH synthesis, and it has been found that erastin treatment depletes intracellular GSH [77–79]. GPx4 inhibits lipid peroxidation by catalyzing the transformation of lipid hydroperoxides into lipid alcohols, utilizing glutathione downstream of the xCT system. RLS3 induces ferroptosis by directly inhibiting GPx4 [80].

Iron is an essential component for ferroptosis, and it is thought that iron chelators block ferroptosis because iron plays the role of electron donor to oxygen for ROS formation. For this, iron must be free and in its reactive form (labile iron pool). A labile iron pool is distributed mainly in lysosomes and the cytoplasm, and it is supplied by circulating iron through the following steps. Protein transferrin receptor 1 (TFR1) imports ferric iron Fe^+3^ (carried by transferrin). It is located in the endosome where it is reduced to ferrous iron Fe^+2^ by the STEAP3 enzyme and released by divalent metal transporter 1 (DMT1) into the cytoplasm iron labile pool. Iron is stored by ferritin, and ferroportin exports it. Ferroptosis-sensitive cells bearing a Ras mutation have increased TFR1 and decreased ferritin; this is an example of iron transport protein deregulation that leads to iron overload. Under this circumstance, excessive Fe^+2^ will produce ROS by the Fenton reaction generating hydroperoxides or lipid hydroperoxides which damage intracellular structures such as membranes [81–83]. This lipid oxidation is inhibited by iron chelator deferoxamine; antioxidants like *α*-tocopherol, GSH, and NAC; and specific ferroptosis inhibitors like ferrostatin-1 (Fer-1), Liproxstatin-1, and Zileuton. Studies with these inhibitors showed that iron-dependent ROS and lipid peroxidation are necessary for ferroptosis induction [84].

Besides iron, ferroptosis also relies on disruption of the antioxidant pathways, for instance, through inhibition of GPx4, the xCT system, and heme-oxygenase-1. One study showed that hemin treatment in human monocytic THP-1 cells induced ROS generation and cell death in a dose-dependent manner. Pretreatment with antioxidants NAC and diphenyleneiodonium chloride (NADPH oxidase inhibitor) as well as the iron chelator deferoxamine (DFO) decreased ROS generation and cell death. Fer-1 decreased hemin-induced cell death, and a combination of hemin and erastin further increased cell death. In addition, neither necroptosis inhibitors nor pan-caspase zVAD-fmk affect cell death rates of hemin-treated cells [85]. Another study also showed the involvement of NADPH oxidase in ferroptosis. SH-SY5Y dopaminergic cells were treated with two pesticides, paraquat and maneb, and results showed induction of ferroptosis associated with NOX. Furthermore, an increase in lipid peroxidation and reductions in GSH and GPx4 were found. Inhibition of NOX with apocynin or diphenyleneiodonium reduced lipid peroxidation, restored GSH and GPx4, and reduced ferroptotic cell death. In addition, NOX activation with phorbol myristate acetate or addition of hydrogen peroxide amplified the effects of both pesticides on ferroptosis [86]. A study in carbon tetrachloride- (CCl4-) induced mouse liver fibrosis showed the presence of ferroptosis biomarkers in fibrotic cells when treated with artesunate. It also showed that artesunate, in human hepatic stellate cell line LX2, induced cell death after 24 hr. Iron accumulation, an increase in ROS production, an increase in lipid peroxidation, and a decrease in GSH content and GPx4 activity, were also observed in these cells, indicating that artesunate triggered ferroptosis *in vitro* and *in vivo.* Furthermore, cotreatment with DFO inhibited iron release, lipid peroxidation, and ROS and increased cell viability compared with artesunate alone in the cell line [87]. Taken together, ROS play a central role in ferroptosis.

### 4.3. Autophagy-Mediated Cell Death

Autophagy is a double-edged sword providing cell survival but under certain conditions, it induces cell death. Autophagy is activated in the context of nutrient and growth factor deprivation, endoplasmic reticulum stress, bacteria infection, and protein aggregate and damaged organelle elimination. It also has a role in cell differentiation, development, growth control, remodelling tissue, among others [88, 89].

There are at least three types of autophagy: chaperon-mediated autophagy, microautophagy, and macroautophagy (hereafter referred to as autophagy, which is the best studied among the three forms). The genes involved in the execution and regulation of autophagy are called autophagy-related genes (ATG), and the resultant proteins form complexes that regulate the different stages of autophagy such as nucleation, autophagosome membrane elongation, autophagosome closure, autophagosome-lysosome fusion, and autolysosome content degradation. The nucleation complex is the structure that forms the membrane autophagosome. The endoplasmic reticulum, mitochondria, Golgi, and plasma membrane are sources for the autophagosome formation, but they also recruit and assemble the complex of vsp34, beclin1, vsp15, and ATG9 in the autophagosome membrane formation. ATG7, the ATG5/12 complex, conjugation of the light chain protein 3 (LC3), and phosphatidyethanolamine are responsible for the autophagosome membrane elongation, and finally, the autophagosome fuse with a lysosome to form the autolysosome. Once in the autolysosome, the cargo will be degraded by hydrolases and the acidification of autolysosome lumen by the proton pump. The constituent components of the degraded cargo exit the lysosome through permeases at the lysosomal membrane into the cytosol. During selective autophagy, proteins like p62 or neighbour of BRACA1 gene 1 (NBRI) act as receptors and adaptors of ubiquitinated substrates to be degraded; hence, they act as bridges between the specific cargo to be degraded and the autophagic machinery [90–93].

Autophagy-mediated cell death is induced through this machinery degrading essential cellular structures to a point where the cell cannot recover. In addition, autophagy could lead to apoptosis through the intrinsic pathway. Under severe stress or injury, cells undergo a form of autophagy-induced cell death called autosis. This autophagy-dependent nonapoptotic form of cell death is characterized by enhanced cell substrate adhesion, focal ballooning of the perinuclear space, and dilation and fragmentation of the endoplasmic reticulum. Autosis is mediated by the Na/K+ ATPase and has been associated with neonatal cerebral hypoxia ischemia [94].

Autophagy is regulated by several negative regulatory pathways. The best characterized mechanism of autophagy regulation is mTOR dependent. Protein kinase mammalian target of rapamycin (mTOR) is an autophagy negative regulator and responds to nitrogen levels in the cell. When nutrients are sufficient, mTOR is activated and autophagy is inhibited. There are several mTOR downstream targets that regulate autophagy. For instance, mTOR complex 1 phosphorylates and actively sequesters the mammalian homolog of Atg1, ULK1, in a complex with Atg13 and FIP200 in an inactive state inhibiting autophagy. AMP-activated protein kinase (AMPK) inhibits mTOR activity, reducing ULK1 phosphorylation and promoting its release from mTORC1, during nutrient deprivation. AMPK can also phosphorylate and activate Tuberous sclerosis complex 2 (TSC2) and Raptor inhibiting of mTOR activity. This in turn activates autophagy [90]. Further downstream, the antiapoptotic protein BCL-2 binds to Beclin-1 preventing the formation of autophagosomes. Under starvation conditions, BCL-2 dissociates from Beclin-1 through JNK phosphorylation of BCL-2 allowing autophagy activation. The growth factor receptor EGFR also binds to Beclin-1 also preventing the induction autophagy. Under cellular stresses such as hypoxia, EGFR is degraded allowing Beclin-1 to induce autophagy. When this negative regulation is eliminated, cells die through an autophagy mechanism [95–97].

Autophagy-mediated cell death also requires ROS. For example, a study showed that macrophages treated with lipopolysaccharides and the pan-caspase inhibitor zVAD induced PARP activation downstream of ROS accumulation inducing autophagic cell death. Another study in our laboratory showed that mitochondrial complex I inhibitor rotenone and complex II inhibitor TTFA induced autophagic cell death mediated by ROS production in HEK 293, U87, and HeLa cells but not in nontransformed cells [98, 99]. Bufaline treatment in colorectal cancer cell lines induced ROS generation, JNK activation, autophagy, and cell death. The NAC antioxidant inhibited JNK activation and decreased autophagic cells and cell death. Also, inhibition of JNK partially decreased autophagic cells and cell death. This study concluded that ROS were responsible for JNK activation which in turn induced autophagic cell death [100]. It has been shown that treatment with amyloid-*β* peptide A*β*_1−42_ in human neuroblastoma cell line SH-SY5Y induced both apoptosis and autophagy, but in the human glioma cell line, U87 induced only autophagy. In both cell lines, A*β*_1−42_ induced production of ROS; however, ROS levels were higher in U87 cells. The NAC antioxidant inhibited autophagy, and autophagy inhibitor 3-MA and NAC inhibited cell death. In contrast, although pan-caspase inhibitor zVAD-fmk prevented the apoptosis induced by A*β*_1−42_ in SH-SY5Y cells, it failed to rescue cells from death. Finally, downregulation of beclin-1 inhibited A*β*_1−42_-induced autophagic cell death, demonstrating that A*β*_1−42_ induced autophagic cell death mediated by ROS [101]. One more study in the U87 cell line showed that treatment with *β*-lapachone-induced autophagy and cell death. Furthermore, neither pan-caspase inhibitor zVAD-fmk nor necrosis inhibitor Necrostatin-1 were able to block cell death; however, autophagy inhibitors 3-MA and bafilomycin as well as siRNA-mediated knockdown of ATG6 or ATG7 expression inhibited cell death. *β*-Lapachone treatment also induced ROS production, and treatment with NAC, *α*-tocopherol, or trolox decreased cell death and autophagy, suggesting that ROS-mediated *β*-lapachone-induced autophagic cell death [102]. Taken together, ROS play a role in autophagy-induced cell death.

### 4.4. Necroptosis

Necrosis has been considered an accidental, passive, nonprogrammed, and uncontrolled form of cell death. Necroptosis is at the crossroads between apoptosis and necrosis and is characterized (like necrosis) by cell swelling, nuclear membrane dilation, chromatin condensation, and membrane permeabilization and subsequent release of cell damage-associated molecular patterns and production of inflammatory cytokines triggering inflammation in surrounding tissue. This regulated cell death type is triggered by perturbations of the extracellular or intracellular environment and is detected by death receptors like FAS and TNFR1 [103, 104]. Necroptosis depends on MLKL, RIPK3, and on the kinase activity of RIPK1. Once initiated by TNFR1, RIPK3 is activated by RIPK1 (as long as caspase 8 is inactivated), then RIPK3 and RIPK1 form an amyloid-like signaling complex called necrosome at which point both proteins undergo a series of trans- and autophosphorylations required for MLKL recruitment and necroptosis activation [105–107].

Negative regulators of necroptosis include CHIP, which promotes RIPK1 and RIPK2 ubiquitination; A20, which promotes deubiquitination of RIPK3; PPM1B, which induces dephosphorylation of RIPK3; and AURKA, which inhibits phosphorylation of MLKL; these four regulators act at the necrosome level [108–111]. Finally, some necroptosis inhibitors are necrostatin-1, 3, and 5 (Nec-1, Nec-3, and Nec-5, respectively); the three of them inhibit RIP1 kinase activity [112, 113].

ROS participate in necroptosis regulation. One of studies reported that selenium nanoparticle (SeNP) treatment induced mitochondrial ROS, mitochondrial damage, TNF and IRF1 necroptotic gene overexpression, increase in RIP1 protein expression, and decrease of cell viability. Necroptosis inhibitor necrostatin-1 increased cell viability [114]. Interestingly selenite has been reported to induce apoptotic cell death in the PC3 cell line accompanied by superoxide generation [115]. Other studies on the colorectal adenocarcinoma Caco-2 cell line treated with an alkynyl gold (I) complex showed antiproliferative effects in a dose- and time-dependent manner with the absence of apoptosis markers. It was also found that this treatment causes loss of mitochondrial membrane potential, ROS generation, and increased RIP1 expression levels. Pretreatment with NAC, necrostatin-1, TNFR1 analog, and NF-*κ*B inhibitor SN50 increased cell survival [116]. TNF-*α* treatment with RelA KO and cIAP1/2 DKO MEF cells induced cell death; however, pretreatment with necrostatin-1 blocked TNF-*α*-induced cell death and pretreatment with the antioxidant, butylated hydroxyanisole (BHA), blocked both induced cell death and ROS accumulation, suggesting that TNF-*α*-induced necroptotic cell death is dependent on ROS. In this study, it is also shown that necrostatin-1 but not BHA inhibited phosphorylation of RIPK1, which suggests that ROS act downstream of RIPK1 activation regulating necroptois [117].

### 4.5. Interplay between ROS and Cell Death

In the studies presented above, the increase in ROS levels is not simply a consequence of cell death but rather a key player in the induction of cell death. These examples showed that the use of antioxidants (scavengers) not only decreased levels of ROS but also rescued cells, and even more, the use of selective ROS inhibitors enlightened us on the particular type of ROS that triggered cell death as shown in Moreno-Manzano et al. In addition to ROS inhibitors, other strategies were used (siRNA, overexpression, transgenic mice) in order to prove that increased ROS or dysfunctional antioxidant systems were directly responsible for cell death induction. These examples, with the use of a combination of ROS inhibitors and cell death inhibitors, also showed the relationship between ROS and cell death. For example, some studies used NAC which promoted not only the decrease in ROS levels but also a decrease in dell death, and then the different cell death inhibitors like zVAD, Fer-1, 3MA, and/or Nec-1 were tested in order to determine the type of cell death induced by ROS. These studies also showed how increased levels of ROS can be caused by either a decrease in antioxidant levels, such as a decrease in GSH or SOD activity [64] or by enhanced ROS production, for instance, by NOX [69] or both [87]. It is worth noting that even though the types of ROS that induce ferroptosis are well defined (iron-dependent ROS and lipid peroxidation), the source of these ROS is not specific for ferroptosis and, it can be shared between different types of cell death; for example, NOX produced ROS that can induce apoptosis [69] or ferroptosis [86]. Another important concept presented in the previous studies is how one stimulus can increase ROS, which in turn will activate two or more different cell death pathways within the cell, but in some cases only one of those two pathways is responsible for causing cell death [101], and in other cases, both or more cell death mechanisms are responsible for cell demise [118] (see below).

### 4.6. ROS and Cell Death Crosstalk

For a long time, the different cell death types (apoptosis, autophagy, ferroptosis, and necroptosis) were studied as independent processes; however, a huge amount of evidence shows that these different types of cell death often share, for example, signaling pathways or initiator molecules and this promotes an interplay between pathways. Since ROS plays a critical role in different forms of programmed cell death, it is not surprising that it could also play a role in crosstalk between these types of cell death. For instance, a study in pancreatic cell lines showed that TRAIL treatment increased ROS and induced apoptosis in the TRAIL-sensitive pancreatic cancer cell lines MiaPaCa-2 and BxPC-3. Inhibition of peroxide decreased apoptotic cell death in MiaPaCa-2 cells but had no effect on BxPC-3 apoptotic cells. On the contrary, superoxide anion inhibition increased apoptosis levels on BxPC-3 cells but had no effect on MiaPaCa-2 apoptosis. In addition, peroxide inhibition increased the necrotic-like population in both cell lines while superoxide inhibition had the same effect but only in BxPC-3 cells. Moreover, addition of necrostatin 1 decreased the necrotic population in both cell lines. To further confirm these results, RIP3 siRNA in combination with TRAIL and peroxide inhibitor in BxPC-3 cells decreased the necroptotic population and increased the apoptotic population. Interestingly, MiaPaCa-2 cells do not express RIP3, only RIP1. These results suggested that TRAIL treatment of MiaPaCa-2 and BxPC-3 cells under inhibition of peroxide promotes RIP1-dependent necroptosis, and that TRAIL treatment under inhibition of ROS (peroxide and superoxide) promotes RIP3-dependent necroptosis in BxPC-3 cells [119].

Combined lysosomotropic agents (siramesine and clemastine) and tyrosine kinase inhibitors (lapatinib and ibrutinib) induced synergistic cell death; however, the type of ROS produced and the type of cell death induced were different depending on the cell type. In breast and lung cancers and in glioblastoma, the combination induced ferroptotic cell death, while in CLL cells, it induced apoptosis. In glioblastoma, LMP leads to an increase in reactive iron, and this leads to iron-dependent oxidation and lipid peroxidation accompanied by a decrease in HO-1 and its antioxidant properties, thus leading to ferroptosis [120, 121]. In contrast, in CLL, LMP leads to an increase in ROS and a loss of mitochondrial membrane potential accompanied by a decrease in antiapoptotic MCL-1, which results in apoptotic cell death [72]. In another study mentioned above, selenium nanoparticles (SeNPs) induced necroptosis in prostate cancer PC3 cells. Interestingly, glucose-decorated selenium nanoparticles induced apoptosis in the hepatocellular carcinoma cell line HepG2. In both cases, the treatment caused mitochondrial damage mediated by ROS [114, 122]. While these studies show how ROS function as a rheostat, the regulatory features of this crosstalk and how ROS controls it remains unclear.

## 5. ROS and Cell Death in Disease

The setting of the ROS rheostat can maintain cellular homeostasis through balancing cell survival with different types of cell death. When this ROS rheostat balance is altered, it can contribute to the development and progression of disease. The link between ROS and cell death and its connection to several diseases is discussed below.

### 5.1. Alzheimer's Disease

Analysis of *post mortem* tissue from Alzheimer's disease (AD) patients and AD models have shown, on one hand, increased ROS and elevated markers of oxidative stress, and on the other hand, the activation of caspase 3, caspase 8, and caspase 9 in sporadic AD [123–127]. One characteristic of AD is the presence of amyloid beta (A*β*) plaques composed of A*β* peptide. Protein and lipid oxidation in brain regions rich in A*β* in early stages of the disease has been found [128]. A*β* peptides produce ROS in the presence of metal ions; similarly, mitochondria also generate ROS causing mitochondrial dysfunction which has been involved in AD pathogenesis [129–131]. The idea that mitochondrial ROS induce mitochondrial dysfunction comes from the “mitochondrial cascade hypothesis” proposed by Swerdlow and Khan. Briefly, it proposes that mitochondrial function and its ability to counteract and recover from stress mediated, among others, by ROS comes from inherited mutations in mtDNA. When oxidative damage amplifies ROS production, three events are triggered: (1) a reset response in which cells respond to elevated ROS by generating A*β* which further perturbs mitochondrial function, (2) a removal response in which compromised cells are purged via programmed cell death mechanisms, and (3) a replace response in which neuronal progenitors unsuccessfully attempt to reenter the cell cycle, thus giving place to A*β* plaques and neurofibrillary tangles [126]. This indicates that ROS regulation is a key feature in AD and may play an important role in future treatments for this disease.

### 5.2. Kidney Failure

Kidney failure is a serious chronic condition. One study showed that acyl-CoA synthetase long-chain family member 4 (*Acsl4*) is a predictive and pharmacodynamic biomarker of ferroptosis *in vivo* in acute kidney failure. In addition, *in vitro* experiments showed that deletion of *Acsl4* caused resistance to erastin and RSL3 induced ferroptosis; however, cells underwent necroptosis when treated with a combination of TNF-*α* and zVAD-fmk. Inversely, *Mlkl*-knockout cells were protected against necroptosis induced by the combination but died by ferroptosis when treated with erastin and RSL3. These observations were confirmed in an *in vivo* model of ischemia-reperfusion injury. ACSL4 protein expression significantly increased during the first 24 hours after reperfusion in *Mlkl*-knockout animals, suggesting a switch toward ferroptosis signaling when necroptosis signaling fails. Even more, an increase in ACSL4 expression in kidney biopsies from patients with acute tubular injury (ATI) was observed following kidney transplantation and severe thrombotic microangiopathy of native kidney [132]. This suggests that limiting lipid peroxidation can inhibit ferroptotic cell death in kidney failure, and crosstalk between ferroptosis and necroptosis in a disease context occurs.

### 5.3. Cancer

One of the hallmarks of cancer is the avoidance of cell death. This occurs through overexpression of antiapoptotic BCL-2 family members and mutations in tumor suppressor genes. In addition, the antioxidant pathways are overexpressed. Collectively, these changes in cancer cells block ROS production in cancer cells to levels that will damage cellular structures leading to cell death. Many chemotherapies selectively increased ROS beyond this limit to induce different types of cell death. Radiation therapy also generates free radicals that damage cellular membranes and DNA leading to apoptosis. Drug resistance prevents apoptosis by protecting the mitochondria from inducing ROS and releasing mitochondrial proteins. Cells also upregulate antioxidant defences limiting ROS levels after cells are treated with chemotherapy or radiation. Immunotherapy for cancer also allows cancer cells to die from ferroptosis where iron-generated ROS plays a critical role [133, 134].

There are several examples where increased ROS induce two or more different types of cell death in different cancer *in vivo* models. For instance, it has been shown that the immunosuppressant FTY720 can induce autophagy, extrinsic apoptosis, and necroptosis in glioblastoma cells. It was also found that autophagy was the promoter of apoptosis and necroptosis and that upstream autophagy, FTY720, inhibited the PI3K/Akt/mTOR/p70S6K signaling pathway by dephosphorylation of Akt, mTOR, and p70S6K. Furthermore, an increase in ROS and the activation of p53 and JNK were found. Conversely, inhibition of p53 and JNK reduces ROS generation, and antioxidant NAC inhibited both p53 and JNK. This suggests the existence of a ROS-JNK-p53 feedback loop. It was also found that this loop participated in the activation of autophagy, apoptosis, and necroptosis. Finally, it was found that FTY720 induced autophagy, apoptosis, and necrosis in xenograft mouse models of human U251 or U87 glioblastoma cells; treatment also inhibited tumor growth, and the high-dose group resulted in smaller tumors compared to the low-dose group [118].

There are few studies *in vivo* showing ROS as a rheostat for one cell death type or another. One exception is studies involving BAY 87-2243, a mitochondrial complex I inhibitor. It was shown that in melanoma cell lines, the inhibitor induced mitochondrial membrane depolarization and increased ROS reduced ATP levels and cell death, which was reversed by vitamin B and glucose. Moreover BAY 87-2243 was able to reduce tumor growth in both BRAF mutant melanoma mouse xenografts and patient-derived melanoma mouse models [135]. It was later found that in melanoma cell lines G361 and SK-MEL-28, BAY 87-2243 induced a combination of ferroptotic/necroptotic cell death due to an opening of the mitochondrial permeability and a decrease in the mitochondrial membrane potential which in turn induced autophagosome formation, mitophagy, and an increase in ROS. zVAD-FMK, a caspase inhibitor failed to prevent cell death; however, necrostatin-1 and ferrostatin-1 both decreased cell death induced by BAY 87-2243. Furthermore RIPK1, MLKL, and GPx4 knockdown prevented loss of cell viability; in contrast, GPx4 overexpression inhibited the BAY-induced increase in intracellular ROS and lipid peroxidation and inhibited reduction in cell viability [136]. Interestingly, in hepatoma cell line Hep3B, treatment with BAY 87-2243 had antiproliferative effects and induced cell death by apoptosis; it was also observed that both effects were enhanced when BAY 87-2243 was combined with histone deacetylase inhibitors [137]. This mitochondrial complex-1 inhibitor was the object of a phase I clinical trial (NCT01297530).

Taken together, cancer cells have the capacity to adapt to toxic ROS levels and different ROS types and to overcome apoptosis through altering the ROS rheostat. In addition, cancer cells can switch from one cell death mechanism to another using ROS as a rheostat. This could be a valuable therapeutic strategy that should be explored. Thus, more *in vivo* studies need to be done in order to confirm the findings presented *in vitro* (Table 2) in which ROS function as a rheostat between two different cell death types.

## 6. Perspective and Future Questions

ROS play major roles in inducing different types of cell death. One reason ROS induce different types of cell death is the different types of ROS being produced within cells. Ferroptosis is induced through iron-generated ROS, whereas induction of apoptosis happens through hydrogen peroxide and superoxide. We have published that oxidative stress-induced autophagy-mediated cell death is driven by a superoxide. Another reason is the ability of cells to remove ROS through antioxidant pathways. This could be through inhibition of GPx4 in ferroptosis or reduced expression of SOD or catalase during apoptosis. This, however, cannot explain all the variations in cell death observed in cells. For example, mitochondrial-derived ROS is detected in many forms of cell death. Another variable is the cell type undergoing cell death. Leukemia cells will undergo apoptosis, but under the same treatment conditions, a glioma cell line will undergo ferroptotic cell death.

Cell organelles play another important role in regulating ROS induction of different types of cell death. Mitochondria damage precedes cell death often caused by ROS but leads to different types of cell death. For example, glucose-coated selenium nanoparticle (SeNP) treatment of prostate cancer leads to necroptosis, whereas SeNP treatment in HepG2 cells induce apoptosis. This could be due to the degree of mitochondrial damage mediated by ROS. Limited damage will release mitochondrial protein leading to activation of the intrinsic pathway, whereas widespread mitochondrial damage will lead to high levels of ROS and lipid oxidation causing plasma membrane damage and necroptosis. Lysosomes are also a source of ROS associated with apoptosis and ferroptosis. Lysosomes are known as a major iron source. Upon lysosome disruption, iron-mediated lipid oxidation and ferroptosis may occur. The same lysosome disruption in other cell types leads to apoptosis not ferroptosis. One possible difference could be differing levels of antioxidant activation allowing for apoptosis before ferroptosis could be induced. Further investigation is warranted to understand how ROS regulates different types of cell death.

There is crosstalk between different types of cell death where players responsible for one type of cell death may play another role in other cell death types. ROS is one of these important players. Indeed, ROS drive different cell death pathways in disease and under treatments. Even though this is not a new concept and several studies have shown this, there are still some questions that cannot be answered with what we know about crosstalk between different cell death types: why the same treatment strategy can induce different types of ROS and in turn different cell death types? And why ROS from the same source induce different cell death types? One way to describe the role of ROS is as a rheostat that allows different types of cell death to be induced under different conditions and with different cell types. How this ROS rheostat is controlled is still not well understood and need to be investigated in this context to predict how cells die and used to develop better treatment strategies for diseases.

## 7. Conclusion

In conclusion, the role of ROS as a rheostat is evident in *in vitro* studies, but its role needs to be further studied *in vivo* to confirm that ROS can act as a rheostat under pathological conditions. Finally, if this phenomenon is proven in different *in vivo* disease models, then the therapeutic potential of this ROS rheostat should be explored.

## Figures and Tables

**Table 1:** Antioxidants and their targets.

Enzymatic antioxidants		
Antioxidant	ROS	Reaction
Superoxide dismutases	Superoxide	O_2_^•-^ + e^−^ + 2H^+^ ⟶ H_2_O_2_
Catalase	Hydrogen peroxide	2H_2_O_2_ ⟶ 2H_2_O + O_2_
Glutathione peroxidase	Hydrogen peroxide	H_2_O_2_+2GSH ⟶ 2H_2_O + GSSG
Thioredoxins	Oxidized proteins	R-S_2_ + Trx-(SH)_2_ ⟶ R-(SH)_2_ + Trx-S_2_
Peroxiredoxin	Hydrogen peroxide	H_2_O_2_ + Prx-S^•^ ⟶ H_2_O + Prx-SOH H_2_O_2_ + Prx-SOH ⟶ H_2_O + Prx-SO_2_H
Nonenzymatic antioxidants		
Antioxidant	ROS	Reaction
GSH	Hydrogen peroxide Oxygen radicals	2GSH + H_2_O_2_ ⟶ GSSG + 2H_2_O GSSG + NADPH + H^•^ ⟶ 2GSH + NADP^+^
*α*^•^-Tocopherol (vitamin E)	Lipid peroxyl radicals	*α*-TOH + LOO^•^ ⟶ *α*-TO^•^ + LOOH *α*-TO^•^ + AscH^-•^ ⟶ *α*-TOH + Asc^•-^
Ascorbic acid (vitamin C)	Free radicals, iron, and copper	AscH^-•^ → Asc^•-^ + 2H^+^ + 2e

**Table 2:** Drugs and type of cell death.

Drug	Model of study	ROS/antioxidant	Cell death type	Ref.
Methylprednisolone	Thymocytes0	Peroxides and decreased GSH	Apoptosis	[71]
Artesunate	Doxorubicin-resistant T leukemia cells	Hydrogen peroxide	Intrinsic apoptosis	[65]
*Vibrio vulnificus* cytolysin	Vascular endothelial ECV304 cells	Superoxide anion	Intrinsic apoptosis	[66]
Angiotensin II	Myocardial ischemia	Superoxide anion and/or hydrogen peroxide	Intrinsic apoptosis	[69]
TNF-*α*	Mesangial cells	Superoxide anion	Extrinsic apoptosis	[65]
Matrine	Liver HL-7702 cells	Hydrogen peroxide and lipid peroxidation Decreased SOD and GSH activity, KEAP1 upregulation, Nrf2 downregulation and inhibition of HO-1 and NQO1	Intrinsic and extrinsic apoptosis	[64]
Oxidized perilla and linseed oil	Neuronal SH-SY5Y cells	Hydrogen peroxide	Caspase-independent apoptosis (AIF) and intrinsic apoptosis	[74]
Clemastine and ibrutinib combination	CLL cells	Superoxide anion and/or hydrogen peroxide	Apoptosis	[72]
Sodium selenite	Prostate cancer PC3	Superoxide anion	Apoptosis	[115]
Glucose-decorated selenium nanoparticles	Hepatocellular carcinoma HepG2 cells	Superoxide anion and/or hydrogen peroxide	Apoptosis	[122]
BAY 87-2243	Hepatoma cell line Hep3B	ROS	Apoptosis	[137]
Siramesine and lapatinib combination	Breast cancer MDA MB 231cells, adenocarcinoma A549 cells, and glioblastoma U87 cells	Iron-mediated ROS and lipid peroxidation	Ferroptosis	[120, 121]
Paraquat and maneb	Neuronal SH-SY5Y cells	Lipid peroxidation NADPH-mediated ROS Decreased GSH and GPx4	Ferroptosis	[86]
Hemin	Monocytic THP-1 cells	Iron-mediated ROS and NADPH-mediated ROS	Ferroptosis	[85]
Artesunate	Hepatic stellate LX2 cells	Iron mediate ROS and lipid peroxidation Decreased GSH and GPx4 activity	Ferroptosis	[87]
Deletion of *Acsl4* and RSL3	Acute kidney failure	Oxidation of fatty acids	Ferroptosis	[132]
Deletion of *Acsl4* and TNF-*α* and zVAD-fmk	Acute kidney failure	Oxidation of fatty acids	Necroptosis	[132]
Rotenone	HEK 293, U87, and HeLa cells	Superoxide anion and/or hydrogen peroxide	Autophagic cell death	[99]
Bufaline	Colon cancer HT-29 and Caco-2 cells	Hydrogen peroxide	Autophagic cell death	[100]
Amyloid-*β*_1-42_	Glioblastoma U87 and SH-SY5Y cells	Hydrogen peroxide	Autophagic cell death	[101]
*β*-Lapachone	Glioblastoma U87 cells	Hydrogen peroxide	Autophagic cell death	[102]
Selenium nanoparticles	Prostate cancer PC3 cells	Superoxide anion and/or hydrogen peroxide	Necroptosis	[114]
Alkynyl gold(I) complex	Colorectal adenocarcinoma Caco-2 cells	ROS	Necroptosis	[116]
TNF-*α*	Mouse embryonic fibroblast RelA KO and cIAP1/2 DKO cells	Hydrogen peroxide	Necroptosis	[117]
FTY720	U87 and U251MG xenograft mouse model	ROS	Autophagy and ferroptosis and necroptosis	[118]
BAY 87-2243	BRAF mutant melanoma mouse xenografts and patient-derived melanoma mouse models	ROS and lipid peroxidation	Ferroptosis and necroptosis	[135]

## References

[1] Clarke P. G. (1990). Developmental cell death: morphological diversity and multiple mechanisms. *Anatomy and Embryology*.

[2] Lettre G., Hengartner M. O. (2006). Developmental apoptosis in *C. elegans*: a complex CEDnario. *Nature Reviews Molecular Cell Biology*.

[3] Clarke P. G. H., Clarke S. (1996). Nineteenth century research on naturally occurring cell death and related phenomena. *Anatomy and Embryology*.

[4] (2008). *Programmed cell death in cancer progression and therapy*.

[5] Galluzzi L., Vitale I., Aaronson S. A. (2018). Molecular mechanisms of cell death: recommendations of the Nomenclature Committee on Cell Death 2018. *Cell Death and Differentiation*.

[6] Mou Y., Wang J., Wu J. (2019). Ferroptosis, a new form of cell death: opportunities and challenges in cancer. *Journal of Hematology and Oncology*.

[7] Hancock J. T., Desikan R., Neill S. J. (2001). Role of reactive oxygen species in cell signalling pathways. *Biochemical Society Transactions*.

[8] Cheeseman K. H., Slater T. F. (1993). An introduction to free radical biochemistry. *British Medical Bulletin*.

[9] Lushchak V. I. (2014). Classification of oxidative stress based on its intensity. *EXCLI Journal*.

[10] (1997). *Oxygen Radicals and the Disease Process*.

[11] (2007). *Encyclopedia of Stress*.

[12] Sies H. (2014). Role of Metabolic H_2_O_2_ Generation:. *The Journal of Biological Chemistry*.

[13] Zhao R., Jiang S., Zhang L., Yu Z.‑. B. (2019). Mitochondrial electron transport chain, ROS generation and uncoupling (review). *International Journal of Molecular Medicine*.

[14] Bak D. W., Weerapana E. (2015). Cysteine-mediated redox signalling in the mitochondria. *Molecular BioSystems*.

[15] Brignac-Huber L., Reed J. R., Backes W. L. (2011). Organization of NADPH-cytochrome P 450 reductase and CYP1A2 in the endoplasmic reticulum—microdomain localization affects monooxygenase function. *Molecular Pharmacology*.

[16] Lüthje S., Möller B., Perrineau F. C., Wöltje K. (2013). Plasma membrane electron pathways and oxidative stress. *Antioxidants & Redox Signaling*.

[17] Vartanian L. S., Gurevich S. M. (1989). NADH- and NADPH-dependent formation of superoxide radicals in liver nuclei. *Biokhimiia (Moscow, Russia)*.

[18] Bedard K., Krause K. H. (2007). The NOX family of ROS-generating NADPH oxidases: physiology and pathophysiology. *Physiological Reviews*.

[19] Lambeth J. D. (2004). NOX enzymes and the biology of reactive oxygen. *Nature Reviews. Immunology*.

[20] Babior B. M., Kipnes R. S., Curnutte J. T. (1973). Biological defense mechanisms. The production by leukocytes of superoxide, a potential bactericidal agent. *J. Clin. Invest*.

[21] Wu R. F., Xu Y. C., Ma Z., Nwariaku F. E., Sarosi G. A., Terada L. S. (2005). Subcellular targeting of oxidants during endothelial cell migration. *The Journal of Cell Biology*.

[22] Zhang A. Y., Yi F., Zhang G., Gulbins E., Li P. L. (2006). Lipid raft clustering and redox signaling platform formation in coronary arterial endothelial cells. *Hypertension*.

[23] Kuroda J., Nakagawa K., Yamasaki T. (2005). The superoxide-producing NAD(P)H oxidase Nox 4 in the nucleus of human vascular endothelial cells. *Genes to Cells*.

[24] Dubois-Deruy E., Peugnet V., Turkieh A., Pinet F. (2020). Oxidative stress in cardiovascular diseases. *Antioxidants*.

[25] Mugoni V., Mattia M., Chai J. (2013). Manipulating redox signaling to block tumor angiogenesis. *Research Directions in Tumor Angiogenesis*.

[26] Che M., Wang R., Li X., Wang H. Y., Zheng X. F. S. (2016). Expanding roles of superoxide dismutases in cell regulation and cancer. *Drug Discovery Today*.

[27] Fukai T., Ushio-Fukai M. (2011). Superoxide dismutases: role in redox signaling, vascular function, and diseases. *Antioxidants & Redox Signaling*.

[28] Glorieux C., Calderon P. B. (2017). Catalase, a remarkable enzyme: targeting the oldest antioxidant enzyme to find a new cancer treatment approach. *Biological Chemistry*.

[29] Goyal M. M., Basak A. (2010). Human catalase: looking for complete identity. *Protein & Cell*.

[30] Brigelius-Flohé R., Maiorino M. (2013). Glutathione peroxidases. *Biochimica et Biophysica Acta Reviews on Cancer*.

[31] Chen Y., Wang K., Zhang D. (2020). GPx6 is involved in the in vitro induced capacitation and acrosome reaction in porcine sperm. *Theriogenology*.

[32] (2019). *Glutathione*.

[33] Lu S. C. (2013). Glutathione synthesis. *Biochimica et Biophysica Acta*.

[34] Lu S. C. (2009). Regulation of glutathione synthesis. *Molecular Aspects of Medicine*.

[35] Nauser T., Koppenol W. H., Gebicki J. M. (2005). The kinetics of oxidation of GSH by protein radicals. *The Biochemical Journal*.

[36] Burton G. W., Traber M. G. (1990). Vitamin E: antioxidant activity, biokinetics, and bioavailability. *Annual Review of Nutrition*.

[37] Traber M. G., Atkinson J. (2007). Vitamin E, antioxidant and nothing more. *Free Radical Biology & Medicine*.

[38] Sies H. (2018). On the history of oxidative stress: concept and some aspects of current development. *Current Opinion in Toxicology*.

[39] (1985). *Oxidative stress*.

[40] NOSE K., SHIBANUMA M., KIKUCHI K., KAGEYAMA H., SAKIYAMA S., KUROKI T. (1991). Transcriptional activation of early-response genes by hydrogen peroxide in a mouse osteoblastic cell line. *European Journal of Biochemistry*.

[41] Devary Y., Gottlieb R. A., Lau L. F., Karin M. (1991). Rapid and preferential activation of the c-Jun gene during the mammalian UV response. *Molecular and Cellular Biology*.

[42] Schreck R., Rieber P., Baeuerle P. A. (1991). Reactive oxygen intermediates as apparently widely used messengers in the activation of the NF-kappa B transcription factor and HIV-1. *The EMBO Journal*.

[43] Reynaert N. L., van der Vliet A., Guala A. S. (2006). Dynamic redox control of NF-kappaB through glutaredoxin-regulated S-glutathionylation of inhibitory kappaB kinase beta. *Proceedings of the National Academy of Sciences*.

[44] Cross J. V., Templeton D. J. (2004). Oxidative stress inhibits MEKK1 by site-specific glutathionylation in the ATP-binding domain. *The Biochemical Journal*.

[45] Kumar Rajendran N., George B. P., Chandran R., Tynga I. M., Houreld N., Abrahamse H. (2019). The influence of light on reactive oxygen species and NF-*к*B in disease progression. *Antioxidants*.

[46] Lingappan K. (2018). NF-*κ*B in oxidative stress. *Current Opinion in Toxicology*.

[47] Fialkow L., Chan C. K., Rotin D., Grinstein S., Downey G. P. (1994). Activation of the mitogen-activated protein kinase signaling pathway in neutrophils. Role of oxidants.. *Journal of Biological Chemistry*.

[48] Simon A. R., Rai U., Fanburg B. L., Cochran B. H. (1998). Activation of the JAK-STAT pathway by reactive oxygen species. *American Journal of Physiology-Cell Physiology*.

[49] Guyton K. Z., Liu Y., Gorospe M., Xu Q., Holbrook N. J. (1996). Activation of Mitogen-activated Protein Kinase by H_2_O_2_:. *Journal of Biological Chemistry*.

[50] Nagai H., Noguchi T., Takeda K., Ichijo H. (2007). Pathophysiological roles of ASK1-MAP kinase signaling pathways. *Journal of Biochemistry and Molecular Biology*.

[51] Zhang J., Wang X., Vikash V. (2016). ROS and ROS-mediated cellular signaling. *Oxidative Medicine and Cellular Longevity*.

[52] Itoh K., Mimura J., Yamamoto M. (2010). Discovery of the negative regulator of Nrf 2, Keap 1: a historical overview. *Antioxidants & Redox Signaling*.

[53] Tong K. I., Katoh Y., Kusunoki H., Itoh K., Tanaka T., Yamamoto M. (2006). Keap 1 recruits Neh 2 through binding to ETGE and DLG motifs: characterization of the two-site molecular recognition model. *Molecular and Cellular Biology*.

[54] Tong K. I., Padmanabhan B., Kobayashi A. (2007). Different electrostatic potentials define ETGE and DLG motifs as hinge and latch in oxidative stress response. *Molecular and Cellular Biology*.

[55] Cuadrado A. (2015). Structural and functional characterization of Nrf2 degradation by glycogen synthase kinase 3/*β*-TrCP. *Free Radical Biology & Medicine*.

[56] Dinkova-Kostova A. T., Holtzclaw W. D., Cole R. N. (2002). Direct evidence that sulfhydryl groups of Keap 1 are the sensors regulating induction of phase 2 enzymes that protect against carcinogens and oxidants. *Proceedings of the National Academy of Sciences of the United States of America*.

[57] Li W., Yu S. W., Kong A. N. T. (2006). Nrf2 Possesses a Redox-sensitive Nuclear Exporting Signal in the Neh5 Transactivation Domain. *The Journal of Biological Chemistry*.

[58] Keum Y.-S., Choi B. (2014). Molecular and chemical regulation of the Keap1-Nrf2 signaling pathway. *Molecules*.

[59] Jung K.-A., Kwak M. K. (2010). The Nrf2 system as a potential target for the development of indirect antioxidants. *Molecules*.

[60] Putcha G. V., Harris C. A., Moulder K. L., Easton R. M., Thompson C. B., Johnson E. M. (2002). Intrinsic and extrinsic pathway signaling during neuronal apoptosis: lessons from the analysis of mutant mice. *The Journal of Cell Biology*.

[61] Sheridan J. P., Marsters S. A., Pitti R. M. (1997). Control of TRAIL-induced apoptosis by a family of signaling and decoy receptors. *Science*.

[62] Yan N., Shi Y. (2005). Mechanisms of apoptosis through structural biology. *Annual Review of Cell and Developmental Biology*.

[63] Ashkenazi A., Dixit V. M. (1998). Death receptors: signaling and modulation. *Science*.

[64] You L., Yang C., Du Y. (2019). Matrine exerts hepatotoxic effects via the ROS-dependent mitochondrial apoptosis pathway and inhibition of Nrf2-mediated antioxidant response. *Oxidative Medicine and Cellular Longevity*.

[65] Moreno-Manzano V., Ishikawa Y., Lucio-Cazana J., Kitamura M. (2000). Selective Involvement of Superoxide Anion, but Not Downstream Compounds Hydrogen Peroxide and Peroxynitrite, in Tumor Necrosis Factor-*α*-induced Apoptosis of Rat Mesangial Cells. *The Journal of Biological Chemistry*.

[66] Hu Q., Wu D., Chen W. (2014). Molecular determinants of caspase-9 activation by the Apaf-1 apoptosome. *Proceedings of the National Academy of Sciences of the United States of America*.

[67] Riedl S. J., Salvesen G. S. (2007). The apoptosome: signalling platform of cell death. *Nature Reviews. Molecular Cell Biology*.

[68] Schriewer J. M., Peek C. B., Bass J., Schumacker P. T. (2013). ROS-mediated PARP activity undermines mitochondrial function after permeability transition pore opening during myocardial ischemia-reperfusion. *Journal of the American Heart Association*.

[69] Erickson J. R., Joiner M. L. A., Guan X. (2008). A dynamic pathway for calcium-independent activation of CaMKII by methionine oxidation. *Cell*.

[70] Marzano C., Gandin V., Folda A., Scutari G., Bindoli A., Rigobello M. P. (2007). Inhibition of thioredoxin reductase by auranofin induces apoptosis in cisplatin-resistant human ovarian cancer cells. *Free Radical Biology & Medicine*.

[71] Fernandez A., Kiefer J., Fosdick L., McConkey D. J. (1995). Oxygen radical production and thiol depletion are required for Ca(2+)-mediated endogenous endonuclease activation in apoptotic thymocytes. *Journal of Immunology*.

[72] Chanas-Larue A., Villalpando-Rodriguez G. E., Henson E. S., Johnston J. B., Gibson S. B. (2020). Antihistamines are synergistic with Bruton's tyrosine kinase inhibiter ibrutinib mediated by lysosome disruption in chronic lymphocytic leukemia (CLL) cells. *Leukemia Research*.

[73] Lorenzo H. K., Susin S. A., Penninger J., Kroemer G. (1999). Apoptosis inducing factor (AIF): a phylogenetically old, caspase-independent effector of cell death. *Cell Death and Differentiation*.

[74] Ueno Y., Kawamoto Y., Nakane Y. (2020). Oxidized perilla and linseed oils induce neuronal apoptosis by caspase-dependent and -independent pathways. *Foods*.

[75] Dixon S. J., Lemberg K. M., Lamprecht M. R. (2012). Ferroptosis: an iron-dependent form of nonapoptotic cell death. *Cell*.

[76] Yang W. S., Stockwell B. R. (2016). Ferroptosis: death by lipid peroxidation. *Trends in Cell Biology*.

[77] Dolma S., Lessnick S. L., Hahn W. C., Stockwell B. R. (2003). Identification of genotype-selective antitumor agents using synthetic lethal chemical screening in engineered human tumor cells. *Cancer Cell*.

[78] Yagoda N., von Rechenberg M., Zaganjor E. (2007). RAS-RAF-MEK-dependent oxidative cell death involving voltage-dependent anion channels. *Nature*.

[79] Sato H., Tamba M., Ishii T., Bannai S. (1999). Cloning and Expression of a Plasma Membrane Cystine/Glutamate Exchange Transporter Composed of Two Distinct Proteins. *The Journal of Biological Chemistry*.

[80] Yang W. S., SriRamaratnam R., Welsch M. E. (2014). Regulation of ferroptotic cancer cell death by GPX4. *Cell*.

[81] Dixon S. J., Stockwell B. R. (2014). The role of iron and reactive oxygen species in cell death. *Nature Chemical Biology*.

[82] Petrat F., de Groot H., Rauen U. (2001). Subcellular distribution of chelatable iron: a laser scanning microscopic study in isolated hepatocytes and liver endothelial cells. *The Biochemical Journal*.

[83] Yang W. S., Stockwell B. R. (2008). Synthetic lethal screening identifies compounds activating iron-dependent, nonapoptotic cell death in oncogenic-RAS-harboring cancer cells. *Chemistry & Biology*.

[84] Xie Y., Hou W., Song X. (2016). Ferroptosis: process and function. *Cell Death and Differentiation*.

[85] Imoto S., Kono M., Suzuki T. (2018). Haemin-induced cell death in human monocytic cells is consistent with ferroptosis. *Transfusion and Apheresis Science*.

[86] Hou L., Huang R., Sun F., Zhang L., Wang Q. (2019). NADPH oxidase regulates paraquat and maneb-induced dopaminergic neurodegeneration through ferroptosis. *Toxicology*.

[87] Kong Z., Liu R., Cheng Y. (2019). Artesunate alleviates liver fibrosis by regulating ferroptosis signaling pathway. *Biomedicine Express*.

[88] Mizushima N., Levine B., Cuervo A. M., Klionsky D. J. (2008). Autophagy fights disease through cellular self-digestion. *Nature*.

[89] Cecconi F., Levine B. (2008). The role of autophagy in mammalian development: cell makeover rather than cell death. *Developmental Cell*.

[90] Singh R., Cuervo A. M. (2011). Autophagy in the cellular energetic balance. *Cell Metabolism*.

[91] Sridhar S., Botbol Y., Macian F., Cuervo A. M. (2012). Autophagy and disease: always two sides to a problem. *The Journal of Pathology*.

[92] Cuervo A. M. (2010). The plasma membrane brings autophagosomes to life. *Nature Cell Biology*.

[93] Lamark T., Kirkin V., Dikic I., Johansen T. (2009). NBR1 and p 62 as cargo receptors for selective autophagy of ubiquitinated targets. *Cell Cycle*.

[94] Liu Y., Levine B. (2015). Autosis and autophagic cell death: the dark side of autophagy. *Cell Death and Differentiation*.

[95] Wei Y., Pattingre S., Sinha S., Bassik M., Levine B. (2008). JNK1-mediated phosphorylation of Bcl-2 regulates starvation-induced autophagy. *Molecular Cell*.

[96] Cui J., Hu Y. F., Feng X. M. (2014). EGFR inhibitors and autophagy in cancer treatment. *Tumor Biology*.

[97] Wei Y., Zou Z., Becker N. (2013). EGFR-mediated Beclin 1 phosphorylation in autophagy suppression, tumor progression, and tumor chemoresistance. *Cell*.

[98] Xu Y., Kim S. O., Li Y., Han J. (2006). Autophagy Contributes to Caspase-independent Macrophage Cell Death. *The Journal of Biological Chemistry*.

[99] Chen Y., McMillan-Ward E., Kong J., Israels S. J., Gibson S. B. (2007). Mitochondrial electron-transport-chain inhibitors of complexes I and II induce autophagic cell death mediated by reactive oxygen species. *Journal of Cell Science*.

[100] Xie C.-M., Chan W. Y., Yu S., Zhao J., Cheng C. H. K. (2011). Bufalin induces autophagy-mediated cell death in human colon cancer cells through reactive oxygen species generation and JNK activation. *Free Radical Biology & Medicine*.

[101] Wang H., Ma J., Tan Y. (2010). Amyloid-*β*1-42 induces reactive oxygen species-mediated autophagic cell death in U87 and SH-SY5Y cells. *Journal of Alzheimer's Disease*.

[102] Park E. J., Choi K. S., Kwon T. K. (2011). *β*-Lapachone-induced reactive oxygen species (ROS) generation mediates autophagic cell death in glioma U87 MG cells. *Chemico-Biological Interactions*.

[103] Vercammen D., Vandenabeele P., Beyaert R., Declercq W., Fiers W. (1997). Tumour necrosis factor-induced necrosis versus anti-Fas-induced apoptosis in L929 cells. *Cytokine*.

[104] Vercammen D., Brouckaert G., Denecker G. (1998). Dual signaling of the Fas receptor: initiation of both apoptotic and necrotic cell death pathways. *The Journal of Experimental Medicine*.

[105] Vandenabeele P., Declercq W., van Herreweghe F., vanden Berghe T. (2010). The role of the kinases RIP1 and RIP3 in TNF-induced necrosis. *Science Signaling*.

[106] Li J., McQuade T., Siemer A. B. (2012). The RIP1/RIP3 necrosome forms a functional amyloid signaling complex required for programmed necrosis. *Cell*.

[107] Cho Y., Challa S., Moquin D. (2009). Phosphorylation-driven assembly of the RIP1-RIP3 complex regulates programmed necrosis and virus-induced inflammation. *Cell*.

[108] Gyrd-Hansen M. (2017). All roads lead to ubiquitin. *Cell Death and Differentiation*.

[109] Onizawa M., Oshima S., Schulze-Topphoff U. (2015). The ubiquitin-modifying enzyme A20 restricts ubiquitination of the kinase RIPK3 and protects cells from necroptosis. *Nature Immunology*.

[110] Chen W., Wu J., Li L. (2015). Ppm1b negatively regulates necroptosis through dephosphorylating Rip3. *Nature Cell Biology*.

[111] Xie Y., Zhu S., Zhong M. (2017). Inhibition of aurora kinase A induces necroptosis in pancreatic carcinoma. *Gastroenterology*.

[112] Degterev A., Huang Z., Boyce M. (2005). Chemical inhibitor of nonapoptotic cell death with therapeutic potential for ischemic brain injury. *Nature Chemical Biology*.

[113] Degterev A., Hitomi J., Germscheid M. (2008). Identification of RIP1 kinase as a specific cellular target of necrostatins. *Nature Chemical Biology*.

[114] Sonkusre P., Cameotra S. S. (2017). Biogenic selenium nanoparticles induce ROS-mediated necroptosis in PC-3 cancer cells through TNF activation. *Journal of Nanobiotechnology*.

[115] Xiang N., Zhao R., Zhong W. (2009). Sodium selenite induces apoptosis by generation of superoxide via the mitochondrial-dependent pathway in human prostate cancer cells. *Cancer Chemotherapy and Pharmacology*.

[116] Mármol I., Virumbrales-Muñoz M., Quero J. (2017). Alkynyl gold(I) complex triggers necroptosis *via* ROS generation in colorectal carcinoma cells. *Journal of Inorganic Biochemistry*.

[117] Shindo R., Kakehashi H., Okumura K., Kumagai Y., Nakano H. (2013). Critical contribution of oxidative stress to TNF*α*-induced necroptosis downstream of RIPK1 activation. *Biochemical and Biophysical Research Communications*.

[118] Zhang L., Wang H., Ding K., Xu J. (2015). FTY720 induces autophagy-related apoptosis and necroptosis in human glioblastoma cells. *Toxicology Letters*.

[119] Zhang M., Harashima N., Moritani T., Huang W., Harada M. (2015). The roles of ROS and caspases in TRAIL-induced apoptosis and necroptosis in human pancreatic cancer cells. *PLoS One*.

[120] Villalpando-Rodriguez G. E., Blankstein A. R., Konzelman C., Gibson S. B. (2019). Lysosomal destabilizing drug siramesine and the dual tyrosine kinase inhibitor lapatinib induce a synergistic ferroptosis through reduced heme oxygenase-1 (HO-1) levels. *Oxidative Medicine and Cellular Longevity*.

[121] Ma S., Dielschneider R. F., Henson E. S. (2017). Ferroptosis and autophagy induced cell death occur independently after siramesine and lapatinib treatment in breast cancer cells. *PLoS One*.

[122] Nie T., Wu H., Wong K. H., Chen T. (2016). Facile synthesis of highly uniform selenium nanoparticles using glucose as the reductant and surface decorator to induce cancer cell apoptosis. *Journal of Materials Chemistry B*.

[123] Aliyev A., Chen S. G., Seyidova D. (2005). Mitochondria DNA deletions in atherosclerotic hypoperfused brain microvessels as a primary target for the development of Alzheimer's disease. *Journal of the Neurological Sciences*.

[124] Khan S. M., Cassarino D. S., Abramova N. N. (2000). Alzheimer’s disease cybrids replicate beta-amyloid abnormalities through cell death pathways. *Annals of Neurology*.

[125] Wang J., Markesbery W. R., Lovell M. A. (2006). Increased oxidative damage in nuclear and mitochondrial DNA in mild cognitive impairment. *Journal of Neurochemistry*.

[126] Rohn T. T., Head E., Nesse W. H., Cotman C. W., Cribbs D. H. (2001). Activation of Caspase-8 in the Alzheimer's Disease Brain. *Neurobiology of Disease*.

[127] Rohn T. T., Rissman R. A., Davis M. C., Kim Y. E., Cotman C. W., Head E. (2002). Caspase-9 Activation and Caspase Cleavage of tau in the Alzheimer's Disease Brain. *Neurobiology of Disease*.

[128] Butterfield D. A. (2014). The 2013 SFRBM discovery award: Selected discoveries from the butterfield laboratory of oxidative stress and its sequela in brain in cognitive disorders exemplified by Alzheimer disease and chemotherapy induced cognitive impairment. *Free Radical Biology & Medicine*.

[129] Opazo C., Huang X., Cherny R. A. (2002). Metalloenzyme-like Activity of Alzheimer's Disease *β*-Amyloid:. *The Journal of Biological Chemistry*.

[130] Rottkamp C. A., Raina A. K., Zhu X. (2001). Redox-active iron mediates amyloid-*β* toxicity. *Free Radical Biology & Medicine*.

[131] Swerdlow R. H., Khan S. M. (2004). A "mitochondrial cascade hypothesis" for sporadic Alzheimer's disease. *Medical Hypotheses*.

[132] Müller T., Dewitz C., Schmitz J. (2017). Necroptosis and ferroptosis are alternative cell death pathways that operate in acute kidney failure. *Cellular and Molecular Life Sciences*.

[133] Hanahan D., Weinberg R. A. (2011). Hallmarks of cancer: the next generation. *Cell*.

[134] de Sá Junior P. L., Câmara D. A. D., Porcacchia A. S. (2017). The roles of ROS in cancer heterogeneity and therapy. *Oxidative Medicine and Cellular Longevity*.

[135] Schöckel L., Glasauer A., Basit F. (2015). Targeting mitochondrial complex I using BAY 87-2243 reduces melanoma tumor growth. *Cancer & Metabolism*.

[136] Basit F., van Oppen L. M. P. E., Schöckel L. (2017). Mitochondrial complex I inhibition triggers a mitophagy-dependent ROS increase leading to necroptosis and ferroptosis in melanoma cells. *Cell Death & Disease*.

[137] Li Y., Rao M.‑. J., Zhang N.‑. Y., Wu L.‑. W., Lin N.‑. M., Zhang C. (2019). BAY 87-2243 sensitizes hepatocellular carcinoma Hep3B cells to histone deacetylase inhibitors treatment via GSK-3*β* activation. *Experimental and Therapeutic Medicine*.

